# Kyste hydatique primitif du sein

**Published:** 2012-08-13

**Authors:** Rabii Mouslik, Abdellatif Settaf, Yacir Elalami, Hicham Lahnini, Khalid Lahlou, Bouziane Chad

**Affiliations:** 1 Service de Chirurgie B, CHU Ibn Sina, Rabat, Maroc

**Keywords:** Kyste hydatique, sein, échographie, mammographie, hydatid cyst, breast, echography, mammography

## Abstract

Le kyste hydatique du sein est une parasitose rare même dans les pays endémiques. Nous rapportons une nouvelle observation d'une patiente de 30 ans qui présentait une masse du sein gauche. Le diagnostic de kyste hydatique du sein a été évoqué devant les données de l'examen clinique et de la mammographie couplée à l’échographie. Le geste chirurgical a consisté en une kystectomie. L'examen anatomopathologique de la pièce opératoire a confirmé le diagnostic.

## Introduction

Le kyste hydatique est une parasitose due au développement chez l'homme de la forme larvaire du tænia *Echinococcus granulosus* [1]. Les principales localisations sont représentées par le foie et le poumon, la localisation mammaire soit primaire ou secondaire reste rare même dans les pays endémiques [2]. Le diagnostic de cette affection est de plus en plus aisé, grâce aux données épidémiologiques et aux différents moyens d'imagerie. Le traitement de cette localisation est toujours chirurgical. Nous rapportons un nouveau cas de kyste hydatique primitif du sein. A travers cette observation et une revue de la littérature nous voulons souligner l'importance de garder cette lésion à l'esprit, malgré sa rareté, particulièrement dans les pays endémiques.

## Patient et observation

**Figure 1:**
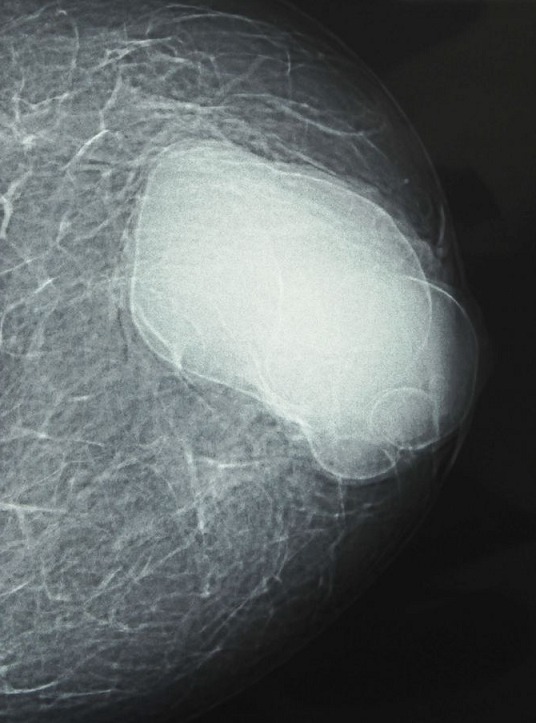
Mammographie montrant une opacité rétro-mamelonnaire gauche, bien limitée et homogène de tonalité hydrique

**Figure 2:**
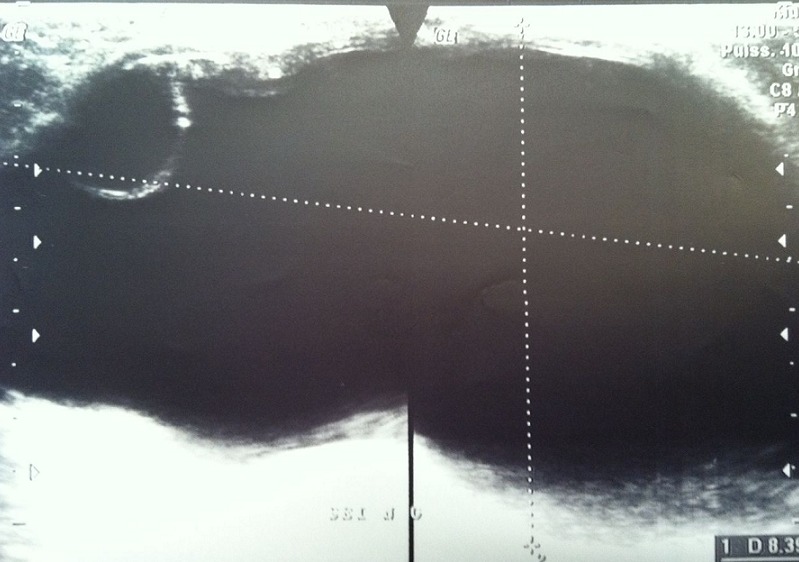
Coupe échographique montrant une lésion kystique rétro-mamelonnaire gauche avec un décollement de membrane

**Figure 3:**
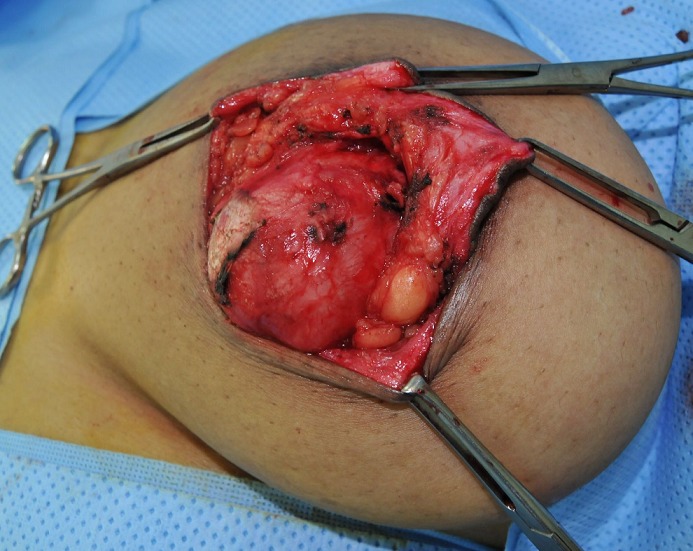
Vue peropératoire du kyste hydatique primitif du sein

**Figure 4:**
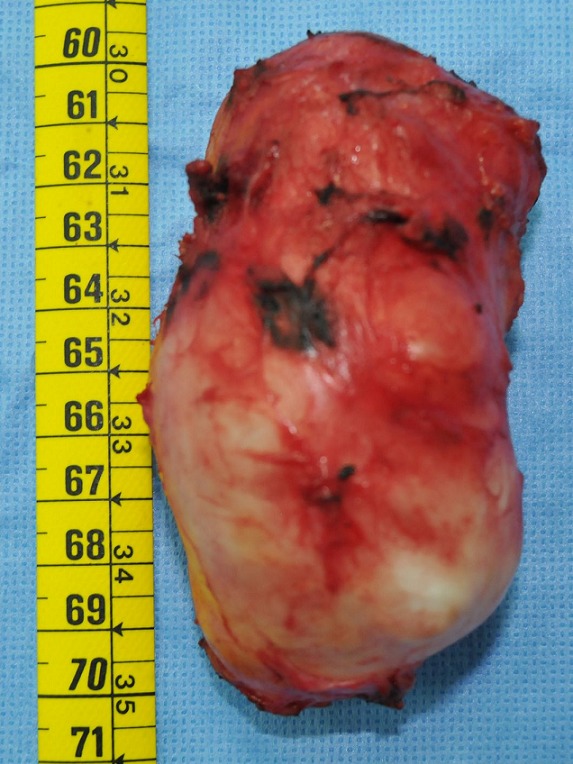
Pièce opératoire du kyste hydatique primitif du sein

Une jeune femme de 30 ans, nulligeste et nullipare, sans antécédents pathologiques particuliers, originaire d'un milieu rural, a consulté pour une masse du sein gauche, évoluant depuis plus d'un an, indolore, augmentant progressivement de taille. L'examen clinique a trouvé une masse du sein gauche rétro-mamelonnaire, mesurant 12 cm de grand axe, bien limitée, mobile par rapport aux deux plans, rénitente, sans signe inflammatoire local. Les aires ganglionnaires étaient libres. Le reste de l'examen clinique était sans particularité. La mammographie avait montré une opacité de tonalité hydrique, homogène, bien limitée de siège rétro-mamelonnaire gauche (Figure 1). L’échographie avait trouvé une volumineuse lésion kystique rétro-mamelonnaire gauche mesurant 85x50mm, à paroi épaissie (3mm), présentant un décollement de membrane par endroits et des vésicules filles (Figure 2). Le sein droit était sans particularité. La radiographie du thorax et l’échographie abdominale n'ont pas montrés d'autres localisations associées. La sérologie hydatique était négative. Le traitement a consisté en une exérèse chirurgicale du kyste par une incision péri aréolaire (Figure 3, Figure 4). L’évolution postopératoire était bonne. L'examen anatomopathologique a confirmé la nature hydatique du kyste. Avec un recul de 1 an aucune récidive n'a été notée.

## Discussion

Le kyste hydatique est une parasitose dont l'agent pathogène est un tænia, *Echinococcus granulosus*, la contamination se fait par voie digestive, de manière directe, par contact avec un chien parasité, ou indirecte par ingestion d'aliments souillés. Le foie est le premier organe atteint. Si le filtre hépatique est dépassé, le parasite est stoppé dans 30 % des cas par le poumon. Ce dernier peut être lui-même dépassé et toutes les autres localisations sont possibles : rate, rein, os, cerveau, parties molles et sein [1, 2]. La localisation hydatique mammaire représente 0,27% de l'ensemble des kystes hydatiques [3], 2,5% des localisations inhabituelles et 0, 3% des tumeurs mammaires [4, 5]. Elle touche essentiellement les femmes de 30 à 50 ans. Notre patiente était âgée de 30 ans. L'hydatidose mammaire est souvent de découverte fortuite lors d'un examen clinique, et ceci du fait de sa latence clinique prolongée qui varie de 2 mois à 20 ans [1]. Cliniquement elle se présente sous forme d'un nodule de consistance rénitente ou ferme, de taille variable, indolore, mobile à contours nets. Il peut prendre l'aspect d'un fibroadénome, une tumeur phyllode, un abcès, voire même un carcinome. C'est pour cette raison qu'il faut l’évoquer devant tout nodule mammaire en zone d'endémie. Le diagnostic repose sur les données épidémiologiques et l'imagerie [4]. La mammographie peut porter le diagnostic en montrant dans les cas typiques, une opacité dense, homogène, bien circonscrite par un liseré de calcifications, ou une opacité arrondie calcifiée dans sa quasi-totalité [5, 6]. Chez notre patiente la lésion correspondait au type I selon la classification d'Ouedraogo qui comprend 4 types [5]. L’échographie est plus contributive que la mammographie dans la démarche diagnostique [7], elle permet de visualiser le kyste quelle que soit sa localisation et définit 5 types selon la classification de Gharbi [1]. Dans notre cas l’échographie a montré une image de type II qui est caractéristique du kyste hydatique. La tomodensitométrie reste le meilleur moyen d'imagerie pour les calcifications périphériques du kyste [8]. L'imagerie par résonnance magnétique peut différencier un kyste hydatique du sein d'une tumeur maligne, mais ne permet pas de faire la différence entre un kyste hydatique surinfecté et un abcès du sein [9]. La cytoponction à l'aiguille fine peut poser le diagnostic en ramenant un liquide eau de roche caractéristique de l'hydatidose. Elle comporte le risque de dissémination voire de choc anaphylactique [9, 10]. Le bilan sanguin peut montrer une hyperéosinophilie qui est non spécifique. La sérologie hydatique est souvent négative ce qui n’élimine pas le diagnostic [10]. La chirurgie reste le seul traitement curatif, elle consiste en une kystectomie en prenant soin de ne pas rompre le kyste [1]. Le traitement par albendazole pendant six mois après la chirurgie est recommandé pour diminuer le taux de récidive [4].

## Conclusion

Le kyste hydatique mammaire, quoique rare, doit être évoqué devant toute masse kystique du sein, surtout en cas d'antécédent de localisation hépatique et dans les pays endémiques. La chirurgie reste le traitement de choix. Les mesures prophylactiques restent le seul garant pour éradiquer cette pathologie.
